# Recent Applications of Mixture Designs in Beverages, Foods, and Pharmaceutical Health: A Systematic Review and Meta-Analysis

**DOI:** 10.3390/foods10081941

**Published:** 2021-08-20

**Authors:** Diego Galvan, Luciane Effting, Hágata Cremasco, Carlos Adam Conte-Junior

**Affiliations:** 1 Center for Food Analysis (NAL), Technological Development Support Laboratory (LADETEC), Federal University of Rio de Janeiro (UFRJ), Cidade Universitária, Rio de Janeiro 21941-598, RJ, Brazil; conte@iq.ufrj.br; 2 Laboratory of Advanced Analysis in Biochemistry and Molecular Biology (LAABBM), Department of Biochemistry, Federal University of Rio de Janeiro (UFRJ), Cidade Universitária, Rio de Janeiro 21941-909, RJ, Brazil; 3 Nanotechnology Network, Carlos Chagas Filho Research Support Foundation of the State of Rio de Janeiro (FAPERJ), Rio de Janeiro 20020-000, RJ, Brazil; 4 Chemistry Department, State University of Londrina (UEL), Rodovia Celso Garcia Cid (PR 445), Londrina 86057-970, PR, Brazil; luciane.effting@uel.br (L.E.); hagata@uel.br (H.C.)

**Keywords:** design of experiments (DoE), experimental designs (EDs), mixture designs (MDs), simplex lattice design (SLD), simplex-centroid design (SCD)

## Abstract

Design of Experiments (DoE) is a statistical tool used to plan and optimize experiments and is seen as a quality technology to achieve products excellence. Among the experimental designs (EDs), the mixture designs (MDs) stand out, being widely applied to improve conditions for processing, developing, or formulating novel products. This review aims to provide useful updated information on the capacity and diversity of MDs applications for the industry and scientific community in the areas of food, beverage, and pharmaceutical health. Recent works were selected following the Preferred Reporting Items for Systematic Review and Meta-Analyses statement (PRISMA) flow diagram. Data analysis was performed by self-organizing map (SOM) to check and understand which fields of application/countries/continents are using MDs. Overall, the SOM indicated that Brazil presented the largest number of works using MDs. Among the continents, America and Asia showed a predominance in applications with the same amount of work. Comparing the MDs application areas, the analysis indicated that works are prevalent in food and beverage science in the American continent, while in Asia, health science prevails. MDs were more used to develop functional/nutraceutical products and the formulation of drugs for several diseases. However, we briefly describe some promising research fields in that MDs can still be employed.

## 1. Introduction

Currently, food and health science studies are becoming more complex with a considerable increase in the number of variables, requiring robust methods for simultaneous data analysis [1]. These analyzes can be facilitated using mathematical and statistical fundamentals, known as chemometric methods, which can be divided into the design of experiments, multivariate data analysis, and multivariate calibration [2,3,4].

Design of Experiments (DoE) is a statistical tool used to plan and optimize experiments and is seen as a quality technology to achieve products excellence [5,6,7,8,9]. Among the various experimental designs (EDs), the mixture designs (MDs) stand out. In MDs, two or more components are mixed in different proportions, and the characteristics of the resulting products are recorded. The responses are independent of physical states, depending only on the proportions of the ingredients present in the mixtures [7,8,10,11].

In MDs, the response depends only on the mixture and not on the mixture’s total amount. The sum of a mixture’s proportions in different components or ingredients is always 1% or 100% [8,12]. In an experiment with *q* components, the proportions of the ingredients may be denoted by *x*_1_, *x*_2_, …, *x_q_*, where *x_i_* ≥ 0 for *i* = 1, 2, …, *q* and $\sum_{i=1}^{q}x_{i}=1$, where *x_i_* represents the proportion of the *i*-th component. This equation removes a degree of freedom from the proportions and the factor space is, therefore, a (*q* − 1)-dimensional regular simplex [2,8,9,13].

A general representation for the MDs is through the figure of an equilateral triangle for systems with three components. The vertices correspond to the pure components and the sides to the binary mixtures, while the points located inside the triangle represent the mixtures of three components, called the central or centroid point [2,8,9]. The variation of a given property with a mixture’s composition is represented by a response surface drawn above the triangle. Thus, the surface’s representation can be done through a triangular diagram of contour lines [2,12].

Over the years, many MDs types have been developed for a specific purpose with extensive application in science, engineering, and industry [2,12]. Among the most common/used are the simplex lattice design (SLD) [9,11,13], simplex-centroid design (SCD) [9,13,14], simplex axial design (SAD) [9,13,15] and extreme vertex design (EVD) [9,13,16,17,18]. For more details, please see the Supplementary Material. Many works that employ designs for experiments with mixture followed the Scheffé regression models [5,6,7,13,19,20,21]. They can be extended to projects with more than three components [9,20]. The widespread models are represented in Equations (1)–(4).

(1) $$\mathrm{Linear}:\;ŷ=\sum_{i=1}^{q}\beta_{i}x_{i}$$

(2) $$\mathrm{Quadratic}:\;ŷ=\sum_{i=1}^{q}\beta_{i}x_{i}+\sum_{i<j}^{q-1}\sum_{j}^{q}\beta_{ij}x_{i}x_{j}$$

(3) $$\mathrm{Special}\;\mathrm{cubic}:\;ŷ=\sum_{i=1}^{q}\beta_{i}x_{i}+\sum_{i<j}^{q-1}\sum_{j}^{q}\beta_{ij}x_{i}x_{j}+\sum_{i<j}^{q-2}\sum_{j<k}^{q-1}\sum_{k}^{q}\beta_{ijk}x_{i}x_{j}x_{k}$$

(4) $$\mathrm{Full}\;\mathrm{cubic}:\;ŷ=\sum_{i=1}^{q}\beta_{i}x_{i}+\sum_{i<j}^{q-1}\sum_{j}^{q}\beta_{ij}x_{i}x_{j}+\sum_{i<j}^{q-1}\sum_{j}^{q}\delta_{ij}x_{i}x_{j}\left(x_{i}-x_{j}\right)+\sum_{i<j}^{q-2}\sum_{j<k}^{q-1}\sum_{k}^{q}\beta_{ijk}x_{i}x_{j}x_{k}$$

The parameter *βi* represents the expected response to the pure blend *x_i_* = 1 and *x_j_* = 0 when *j* ≠ *i*. The term $\sum_{i=1}^{q}\beta_{i}x_{i}$ represents the linear blending portion. When curvature arises from nonlinear blending between component pairs, the parameters *β_ij_*, which represent either synergistic or antagonistic blending, will be different from zero [2,8].

Developing a novel product requires mixing two or more ingredients, and specific characteristics or restrictions are often desired [22]. Therefore, product formulation is still a challenge that has dogged chemists, pharmacists, and food scientists. Conventionally, product development requires much work and experiments, which increase with the number of variables evaluated. Furthermore, when experiments are based on trial and error, without an experimental design, there is the possibility of presenting low reproducibility, robustness, versatility, as they are not statistically validated [23].

In this sense, the use of MDs makes it possible to find the best proportion of these components through predictive equations that allow the application of mathematical algorithms, making it possible to determine the optimal conditions for a formulation or an industrial process [2,5,6,7,8,13,19]. The application of these models aims at high quality, low cost, and optimizes single or multiple responses simultaneously (applying desirability functions), and solves challenges with restrictions [5,6,7,19].

Improvements in food, beverage, and health processing techniques have been increasingly recurrent due to the general expectation for higher quality/quantity products today. Jointly, factors that ensure food security to these demands. Thus, MDs can facilitate the development of novel products or techniques that seek to achieve these goals. Among some typical applications, we can list the development of foods and beverages with functional or nutraceutical properties [22,24,25,26], while in the pharmaceutical health area, they are used to formulate drugs for several diseases [23,27,28]. In addition, MDs can also be used for many other applications [5,29,30].

In this context, this work presents the recent applications of MDs in beverage, food, and pharmaceutical health worldwide. Furthermore, it will be shown which MDs models can be used in these sectors, statistical packages used, behaviors, trends, and perspectives of MDs applications.

## 2. Methodology

Initially, a preliminary selection of scientific papers was carried out through the abstract, keyword, and title content. Papers were then removed in this initial screening if they did not investigate the association between MDs. The search was limited to English, and the date delimitation was set as between 2016 and 2020. Our search was limited to published papers that recently. MDs works between the years 1955 to 2016 have already been reported by Piepel [31] and Sahin and collaborators [32]. Editorials, letters, and Ph.D. thesis were excluded from this review. The results are reported in agreement with the Preferred Reporting Items for Systematic Review and Meta-Analyses statement (PRISMA) flow diagram [33].

### 2.1. Focus Questions

The question was developed according to the population, intervention, comparison, and outcome (PICO) method. In which area is the MDs most applied? How important is each country or continent to the application areas? How are MDs being employed within each area? What are the most common products that use MDs?

### 2.2. Information Sources

Data searches were obtained from online databases PubMed, Scopus, and Web of Science. The screening process was performed in February 2020. Further directed searching was also carried out by checking the reference list of relevant articles. Three research fields were selected in which the use of MDs is common: beverage, food, and pharmaceutical health. Selected papers that used MDs with different applications from the three research fields contained in the online research database were considered and classified as “other” areas.

**Search component 1 (SC1)** included the following population search:

**SC1a–Beverage:** (beverage* OR brew* OR drink* OR refreshment* OR juice* OR alcoholic drink* OR beer* OR yogurt* OR dairy drink* OR distilled beverage* OR fermented beverage* OR soda*).

**SC1b–Food:** (food* OR aliment* OR feed* OR meat* OR cake* OR fruit* OR vegetable* OR fish* OR dairy derivatives* OR animal origin* OR vegetable origin* OR honey* OR milk* OR egg* OR oil* OR edible oil* OR cheese* OR sausage*).

**SC1c–Pharmaceutical health:** (gel* OR emulgel* OR capsule* OR cream* OR ointment* OR packing* OR formulation* OR nano-formulation* OR drug* OR medicament* OR remedy* OR pharmaco*).

**Search component 2 (SC2)** included the following intervention search:

**SC2–Mixture designs:** (mixture design* OR simplex centroid* OR simplex-centroid design* OR mixtures design* OR d-optimization OR desirability function).

After retrieving the search component results, the Boolean operator “AND” to combine SC1a, SC1b, SC1c, and SC2. The asterisk serves as a wildcard operator. In other words, the search engine will highlight any word that begins with the root/stem of the word with the word preceding the * operator.

Finally, the papers were tabulated and separated by each research field, continent, and country to which the authors belonged. In works with authors from different countries, these works were included in both countries to which the authors belonged. In this study, it should also be noted that Russia and Turkey were considered to belong to the continents of Asia and Europe.

### 2.3. Risk of Bias Assessment

Possible sources of bias include study inclusion/exclusion criteria, the chosen database, date, language, number of articles, the impact of missing data, missing primary results, and article type selected for this study [34].

### 2.4. Data Analysis

Self-organizing map (SOM) is a type of artificial neural network (ANN) based on less conventional statistical principles, which does not need in-depth knowledge in statistics and multivariate analysis [35,36]. The Kohonen self-organizing map algorithm begins initializing the first grid with random synaptic weights and no organization applied to the map. Three key processes take place: competition, cooperation, and synaptic adaptation [34,35,36,37,38]. The SOM network routine developed was used according to the algorithm described in Haykin [37] and was applied using the Matlab software routine. For more details about the theoretical and mathematical foundations of the SOM algorithm, readers can consult the references [34,39,40] and Supplementary Material.

ANN was carried out to verify and understand which fields of research and countries/continents are using the MDs. Research fields such as beverage, food, pharmaceutical health, and others for each country were tabulated and used as input variables for the SOM. Three SOM networks were processed, one for the continents and two for the countries.

The SOM network was applied, considering two scenarios. In the first, 52 countries were included. In the second, Brazil was excluded, totaling 51 countries. Brazil was removed due to the large number of works using the MDs. This exclusion allows a better assessment of the use of these tools in other countries. The data sets contained 208 points (4 columns/variables (beverage, food, pharmaceutical health, and others), for 52 rows/countries) for scenario 1. A total of 204 points (4 columns/variables (beverage, food, health, and others) for 51 rows/countries) in scenario 2. For the continents, the input data set contained 20 points (4 columns/variables (beverage, food, pharmaceutical health, and others), for 5 rows/continents). The country names were abbreviated according to the United Nations (UN) codes for better visualization of the data set, presented in Table 1.

SOM setup a was the hexagonal topology of 12 × 12 (for countries) and 4 × 4 (for continents) with 7000 training epochs to ensure a convergence of the mean quantization error (MQE). The neighborhood parameter began with an initial value of 5.5 (countries) and 1.5 (continents), decreasing to 0.03 and 0.04, respectively; the learning rate started at 0.20, decaying exponentially with the training epochs to 2 × 10^−4^ for both cases, with MQE values of 1 × 10^−4^ and 0.02, respectively.

## 3. Mixture Designs Applications

### 3.1. Literature Search

**Figure 1:**
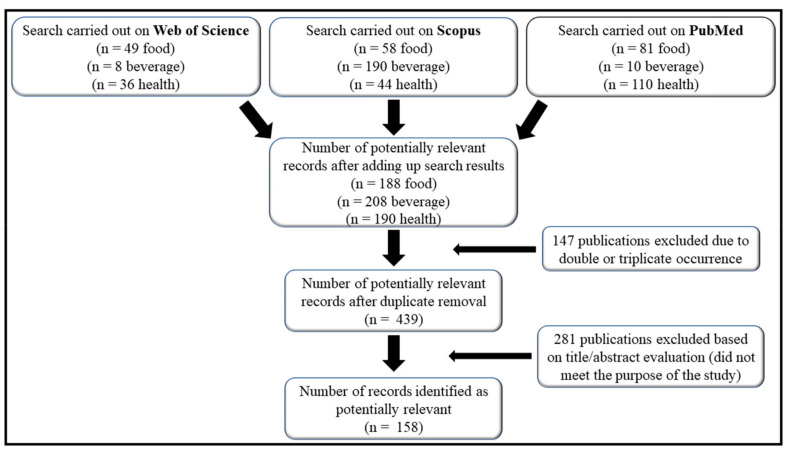
Flow diagram displaying the literature search results at the PubMed, Web of Science, and Scopus databases and overview of the chemometric study.

A total of 586 papers were identified, 292 papers at Scopus (190 beverage, 58 food, and 44 pharmaceutical health), 201 papers at PubMed database (10 beverage, 81 food, and 110 pharmaceutical health), and 93 papers at Web of Science (8 beverage, 49 food, and 36 pharmaceutical health). Each paper was obtained and evaluated individually. Of these, 147 were duplicates or triplicates and were excluded. A total of 439 remained after the exclusion of repeated papers. After screening the titles and abstracts, only 158 papers were adequate for the study (Figure 1).

### 3.2. Application of Mixture Design around the World

**Figure 2:**
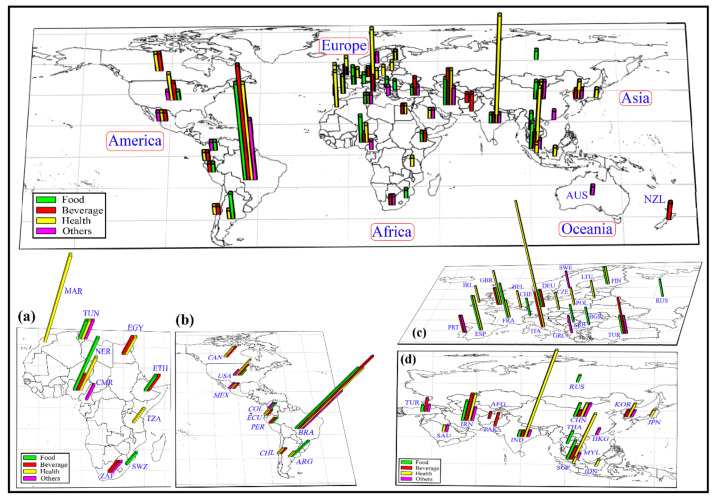
Application of mixture designs around the world between 2016 and 2020. Africa (**a**), America (**b**), Europe (**c**), and Asia (**d**).

Initially, all possible applications for MDs around the world were considered. According to the data set, Figure 2 shows the countries and their continents that most used MDs in the beverage, food, pharmaceutical health, and other science in the last five years.

According to Figure 2, a previous visual analysis shows the prevalence of works with MDs in Brazil, standing out from other countries. However, only visual analysis can omit relevant information easily resolved by introducing appropriate tools, such as SOM, which are great for pattern recognition and classification [36,38]. Visual analysis indicated the prevalence of Brazil; for this reason, the study was separated into two scenarios. In scenario 1, the data were evaluated, including Brazil, and in scenario 2, Brazil was excluded from the analysis. The exclusion of Brazil, in scenario 2, allows better classification and discussion of results for other countries.

**Figure 3:**
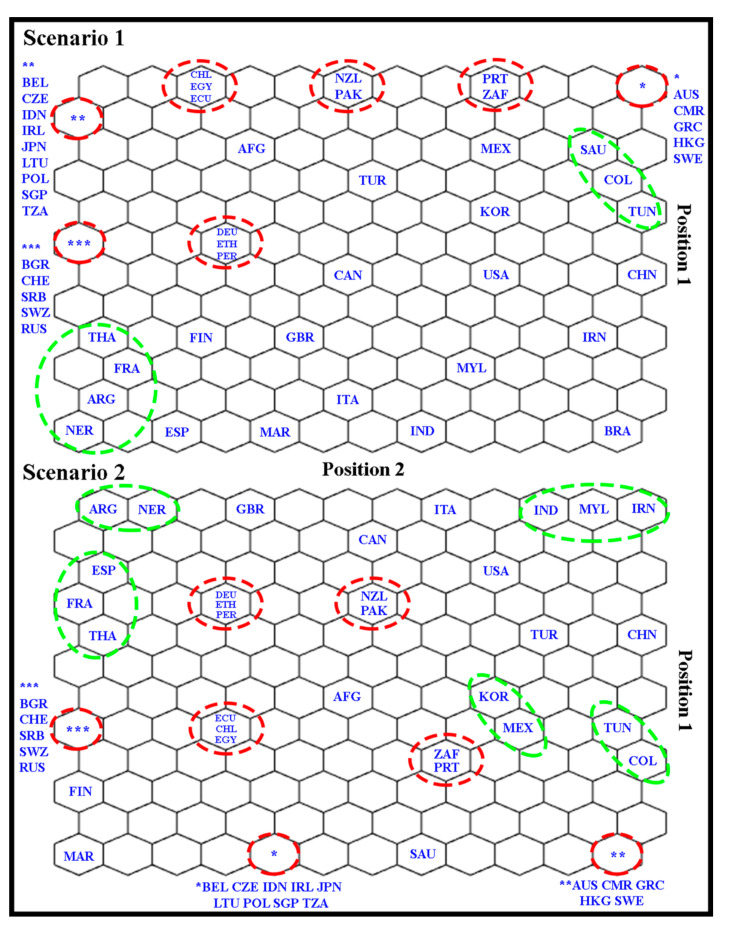
Countries distribution according to the winning neuron for both scenarios.

After the SOM training stage, topological maps were generated, and the countries’ distribution according to the “winning neuron” for the two scenarios were evaluated (Figure 3). In the topological map, each country is associated with a winning neuron, i.e., the one best representing that country in the SOM. The SOM network classifies the input data as clusters that can be formed by one or more neurons. The definition of clusters is characterized by the presence of empty neurons among the groups [36,38].

According to Figure 3, it can be concluded that in both scenarios, there was the formation of clusters, i.e., the SOM network identified that the MDs are used differently in each country. However, only the topological map does not allow to say which area the mixture design is being applied for each country or continent. We will discuss the different areas of MDs applications later with the weight maps.

In Figure 3, the network indicated the formation of 9 clusters for scenario 1, while in scenario 2, the number of clusters increased to 12. These clusters highlighted in red in scenarios 1 and 2 represent the countries classified in the same winning neuron, i.e., these countries have the same importance when using MDs. However, empty neurons between these clusters indicate that these groups of countries have different importance. For example, the cluster from Bulgaria (BGR), Chile (CHE), Serbia (SRB), Eswatini (SWZ), and Russia (RUS) differs more from the cluster from Australia (AUS), Cameroon (CAM), Greece (GRC), Hong Kong (HKG) and Sweden (SWE), when compared to the cluster from Germany (DEU), Ethiopia (ETH) and Peru (PER). This same observation can be made for the other clusters.

Clusters highlighted in green in Figure 3 indicate a certain similarity between these countries, as they have been classified into neighboring neurons, i.e., similarly contribute to the use of MDs. In scenario 1, two clusters were formed, one cluster from Colombia (COL), Saudi Arabia (SAU), and Tunisia (TUN), and another from Argentina (ARG), France (FRA), Nigeria (NER), and Thailand (THA). Regardless of the certain similarity between the countries classified in each cluster, these two clusters have different characteristics. This fact is attributed to the presence of empty neurons between these clusters. Another observation was that Brazil (BRA) remained separate into a single neuron; furthermore, many neighboring empty neurons in the network between them indicated that Brazil stands out from other countries.

In scenario 2, a greater formation of clusters is observed than in scenario 1 (Figure 3). We highlight the formation of five clusters formed by neighboring neurons, where the cluster from Argentina (ARG) and Nigeria (NER) have some similarities with the cluster from France (FRA), Spain (ESP), and Thailand (THA). This same argument is applicable for the cluster from Mexico (MEX), South Korea (KOR), with the cluster from Tunisia (TUN), and Colombia (COL). However, these pairs of clusters differ due to the relationship of empty neurons between them. Moreover, the cluster from India (IND), Iran (IRN), and Malaysia (MLY) formed an isolated cluster, i.e., it is more different from the other groups. It is also observed that in this cluster, there are only countries of the Asian continent.

**Figure 4:**
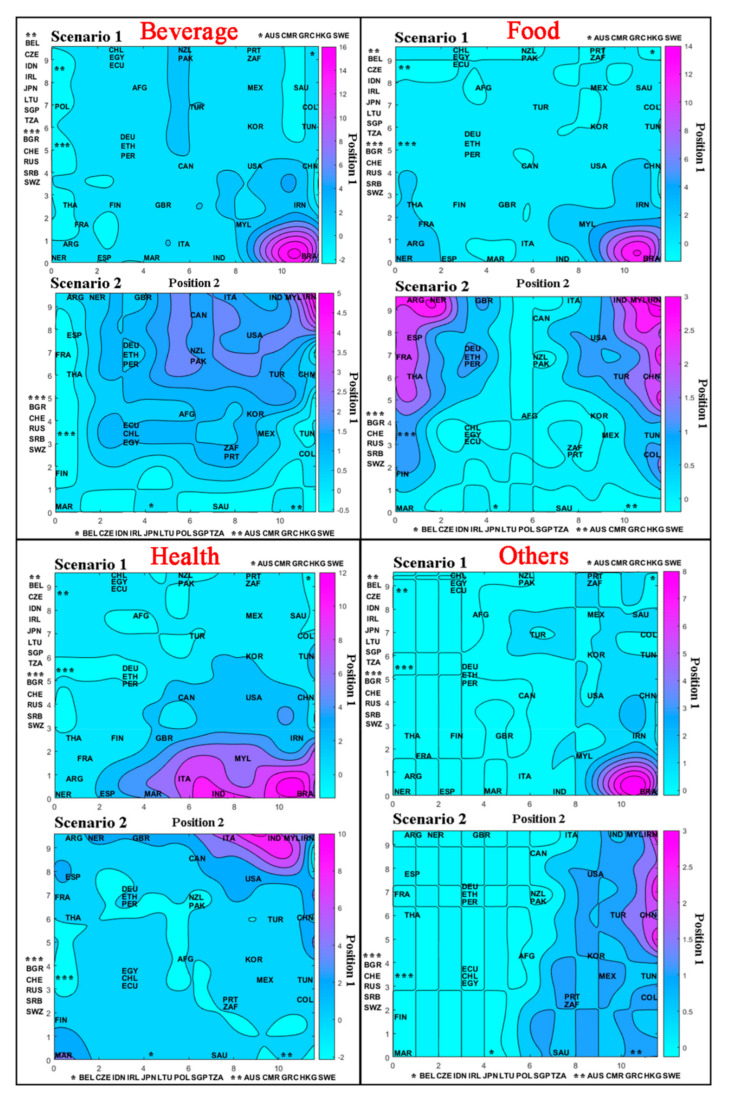
Weight maps overlaid by topological maps for the variables: countries. Position 1 and Position 2 indicate the position of the winning neuron.

After analyzing the topological map that represents the countries distributed in each winning neuron (Figure 3), the respective weight maps for application areas of the MDs were generated, shown in Figure 4. Further discussion for each area will be given in the following sections. The weight maps represent the topological map overlay for the continent’s segmentation, and the color scale indicates the observed values for the input variables.

### 3.3. Application of Mixture Design by Continent

**Figure 5:**
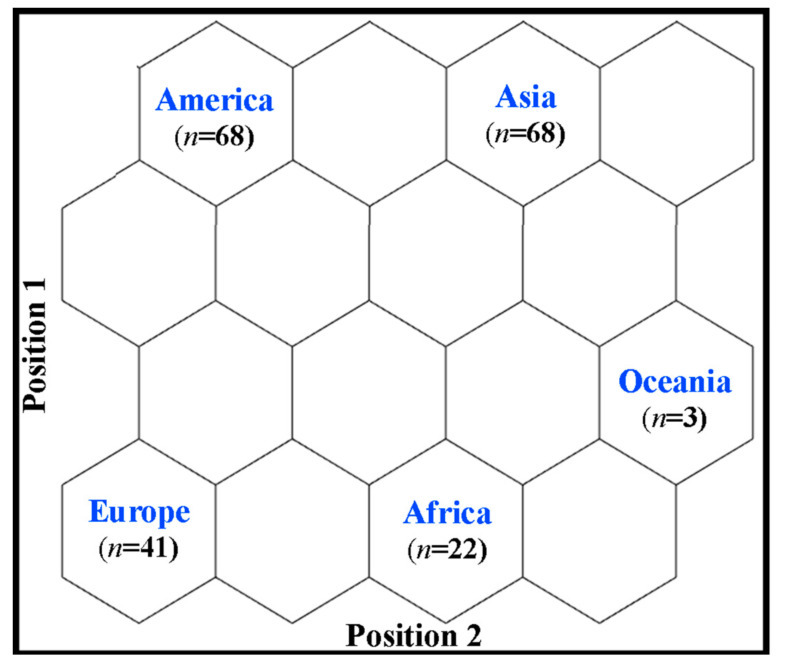
Continents distribution according to the winning neuron.

Topological maps were generated for the distribution of continents according to the winning neuron. According to Figure 5, each of the five continents evaluated has a single winning neuron, i.e., these continents differ from each other concerning the use of MDs. Although it does not form clusters, the relationship of empty neurons between each continent provides relevant information. The SOM analysis indicated that the continents of America and Asia are more like each other and differ from other continents. Moreover, Europe is more like Africa and differs more from America, Asia, and Oceania. The continents of Oceania and Africa resemble each other and differ more from America.

As previously discussed, the continents of America and Asia showed some similarities between them. This behavior is mainly due to the participation of papers published by Brazil (BRA) and the United States (USA) in America, while India (IND), Malaysia (MYL), and Iran (IRN) were the most responsible for Asia. Europe is somewhat similar to Africa; however, it differs from other continents. The similarity between Europe and Africa is mainly due to the works published by Italy (ITA) and Nigeria (NER). When compared to the other continents, Oceania was the continent that presented the lowest number of publications. For this reason, it differs well from America, which presented the largest number of publications.

In Figure 5, the countries with the largest publications are presented at the top of the distribution map, represented by America (*n* = 68) and Asia (*n* = 68). At the bottom of the map, the continents with the lowest quantities, represented by Europe (*n* = 41), Africa (*n* = 22), and Oceania (*n* = 3). In addition, the bottom part of the distribution map indicated that these three continents could be separated decreasingly from left to right.

**Figure 6:**
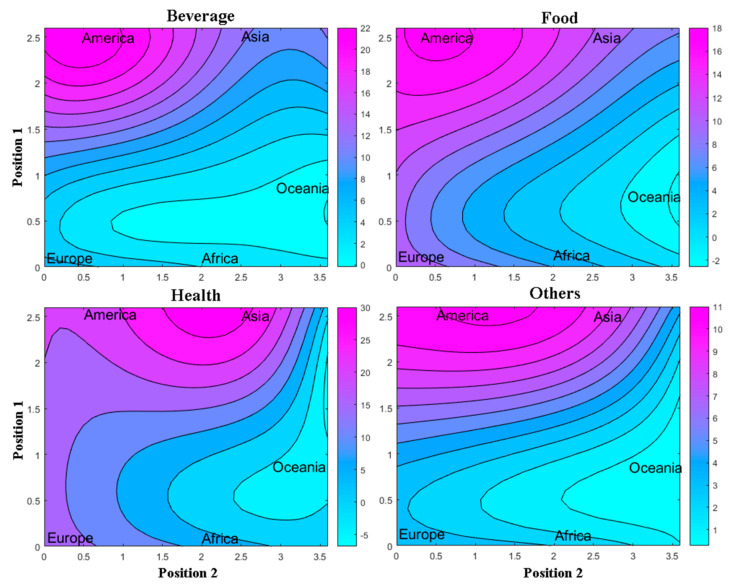
Weight maps overlaid by topological maps for the variables: continents. Position 1 and Position 2 indicate the position of the winning neuron.

Weight maps in Figure 6 illustrate each of the application areas of the MDs by continent, where the American continent is most prevalent. In the Asian continent with applications in the area of health sciences predominate.

### 3.4. Application of Mixture Design by Area

**Figure 7:**
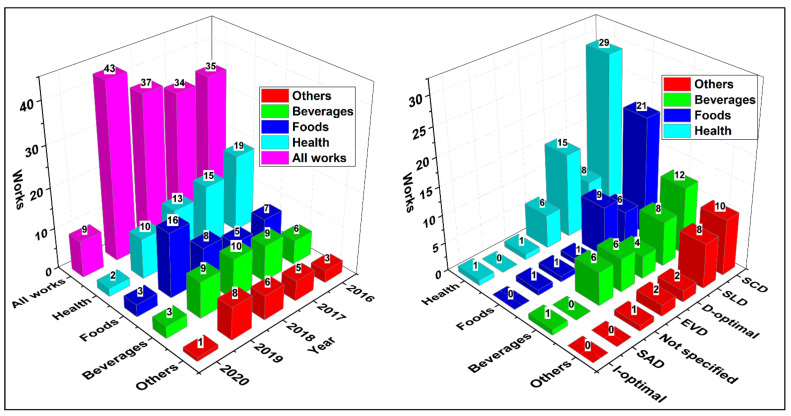
Number of works by areas and year using MDs according to the search from January 2016 to February 2020.

Overall, Figure 7 shows that MDs have been increasingly applied in different areas of knowledge over the years, being more applied in the pharmaceutical health area, followed by the food and beverage areas. In this sense, researchers have applied MDs to improve processing conditions, new product development, or obtain unique sensory and physicochemical characteristics in products, among other numerous applications described briefly in the following sections.

Among the most reported MDs, the simplex-centroid (SCD), simplex lattice (SLD), extreme vertex designs (EVD), and D-optimal designs have been the most frequent. Choosing a specific method is not an easy task. There are countless situations where various modeling methods can be integrated into different MDs types [32]. Moreover, it must be clear that these tools do not replace the specialist’s technical knowledge, who must keep in mind that knowledge of the problem domain is essential to achieve a successful experiment [3].

After obtaining the response, it is necessary to evaluate the data obtained. The simplest way is to evaluate the surface of each response, looking for intersections between the ideal regions of each one. Furthermore, this step does not require much mathematical knowledge, only specific decision criteria in choosing optimal conditions that simultaneously meet the evaluated responses. However, defining the ideal point becomes increasingly difficult as more variables are evaluated, making it more difficult to find a single condition that simultaneously satisfies the entire experimental design [41,42].

Another possibility is to transform each response into a dimensionless scale of individual desirability (*d*_i_), which are then aggregated into a single response called global desirability (D). By adjusting a mathematical model to the values of D, it is possible to simultaneously optimize all variables considering all available responses [5,19,22]. Some examples of its application are briefly discussed in Section 3.9. Another way to assess the quality of the adjusted model is by applying analysis of variance (ANOVA). From this comparison, it is possible to assess the significance of the regression used to predict the responses considering the sources of experimental variance [41,42].

### 3.5. Application of Mixture Design in Beverage

Figure 4 illustrates the weight maps for the beverage variable. In general, when comparing the weight maps for the beverage variable in the two scenarios, the difference is evident when Brazil is included. In scenario 1, Brazil (BRA) presented the largest number of publications using MDs, followed by Iran (IRN), Malaysia (MYL), New Zealand (NZ), and Pakistan (PAK). Other countries such as Germany (DEU), Mexico (MEX), United Kingdom (GBR), among other countries represented by the same color scale, presented smaller quantities (*n* = 1). Poland (POL), Saudi Arabia (SAU), Thailand (THA), among other countries represented by the same color scale, did not present any publication in the beverage area.

In Figure 4 of scenario 1, the American continent had greater participation in the beverage area than the other continents. This behavior is driven mainly by works published by Brazil (BRA), totaling *n* = 13. Followed by the Asian continent, represented mainly by Iran (IRN), Malaysia (MYL), and Pakistan (PAK) works, totaling *n* = 8. Other continents did not present countries with a significant number of publications in this area, except New Zealand (NZL) in Oceania, totaling *n* = 2.

In scenario 2, there was a better differentiation of the countries using MDs (Figure 4), where the upper quadrant on the right side of the weight map showed higher values, i.e., in this region, the countries most use MDs for this application. Iran (IRN), Malaysia (MYL), Pakistan (PAK), Turkey (TUR), Italy (ITA), Canada (CAN), United States (USA), and New Zealand (NZL) are the most representative countries, represented by the Asian continent, European, American and Oceania continents, respectively.

Table 2 shows some products that used MDs to improve the conditions for processing or formulating beverage products, classified into four subdivisions following careful previous analysis: alcoholic beverage, milk-based beverages, several beverages, and several juices.

In **alcoholic beverages**, simplex-lattice MDs was applied to craft beers to understand the sensory contribution of different hops (Cascade, Chinook, and Centennial) added to the American beer style. The study demonstrated that it is possible to achieve similar aroma profiles when dry-hopping beer with varying blends of Cascade, Chinook, and Centennial hops [43]. The use of MDs for these products has been scarce, demonstrating the possibility for future studies that seek to formulate alcoholic beverages with functional or antioxidant properties, as well as evaluate the effects of additions of spices in a beverage at different stages of fermentation, mixtures of raw materials, formulations of gluten-free or non-alcoholic beverages, among several other possible applications [22].

In **milk-based beverages**, the most frequent applications of MDs aim to improve the rheological, functional, sensory, and antioxidant properties of yogurts and fermented beverages based on milk. Probiotic products were most frequently evaluated, such as yogurt [46,48,49], fermented milk [51], and chocolate beverages [47]. Furthermore, other conventional products, such as yogurt [44,50,55] and dairy drink [57] formulation; yogurt [53] and milk chocolate [45] diet or light; fermented milk [52,56] and soy milk-based beverage [54] have been reported.

In this works, the authors use MDs for formulations of yogurt or fermented milk symbiotics, probiotics, or organic [46,49,51], with various applications. Such as investigate the influence of starter cultures mixtures for product development [48,55], evaluate the effects of different gums and their interactions on rheological, physicochemical, and sensory properties [44,47,52,56], formulate beverages with low/free sugar content [53,54], formulation of drinks with natural sweeteners [45], fortified with antioxidant [50], or for the formulation of low-cost milk drinks obtained from by-products [57].

In **several beverages**, the MDs were used to formulate some unconventional beverages, such as the development of a drink based on sweet potato peels, and a tea based on rooibos (*Aspalathus linearis*), white tea (*Camellia sinensis* var. *sinensis*), and roasted mate (*Ilex paraguariensis*) optimizing the antioxidant and antiproliferative activity of beverages [25,64]. As well as other formulated beverages, such as the development of banana, strawberry, and juçara smoothie [62], carbonated soft drinks formulated based on tagatose, sucrose, and stevia [59,61], coffee [60,63], peanut drink [58], and soursop [65] beverage formulation. Among the applications of MDs in several beverages, we highlight the authors’ attempt to achieve improvements in sensory parameters, antioxidant, physical and chemical properties, and rheological of beverages.

In **several juices**, the applications of the MDs were in functional fruit juices [24,67,68,69,70,71,72,74,75,76], powders [66,73], and the quality of sugarcane [77] juice. In addition, the MDs have often been applied in formulations of probiotic and energetic functional juices to improve these products’ sensory, antioxidant, and nutritional properties with potential benefits for consumers’ health [66,68,72,73].

Among the most common uses of MDs in several juices, there were reports of studies that evaluated the reduction in calories in juices [67], the addition of stevia [74], and the inclusion of phenolic and bioactive compounds in juices [71]. Part of these studies focuses on investigating Brazilian native fruits, such as acerola [67], and other fruits native to the Brazilian Cerrado, such as Marolo, Mangaba, and Cagaita [75]. Due to Brazil’s diversity of fruits and the small extent of knowledge of the vast properties these fruits can contain, this may be a probable reason why MDs have been used to formulate functional juices in Brazil.

### 3.6. Application of Mixture Design in Food

Figure 4 shows the weight map of the food variable for the two scenarios. In scenario 1, the food variable presented the same behavior as for the beverage area. In this field, Brazil (BRA) again presented more publications. Other countries with significant amounts of food area publications were Iran (IRN), Malaysia (MYL), China (CHN), Thailand (THA), France (FRA), Spain (ESP), Argentina (ARG), and Nigeria (NER), represented by the Asian, European, American, and African continents, respectively. Peru (PER), Finland (FIN), Colombia (COL), among other countries represented by the same color scale, presented fewer quantities of publications (*n* = 1). Countries such as Afghanistan (AFG), Portugal (PRT), Morocco (MAR), among others represented by the same color scale, did not have work in the area.

Figure 4 of scenario 1 shows that the American and Asian continents had the largest food area participation than the other continents. The largest participation is from Brazil (BRA) and Argentina (ARG) in America, totaling *n* = 14. In Asia, the largest participation is mainly by Iran (IRN), Malaysia (MYL), Thailand (THA), and China (CHN), totaling *n* = 10 papers. Europe and Africa had smaller participation, with France (FRA) and Spain (ESP) being the main countries on the European continent, and Nigeria (NER) the main on the African continent, totaling *n* = 4 and *n* = 3, respectively.

In scenario 2, the weight map presented higher values in the upper left and right quadrant. In these quadrants are Iran (IRN), Malaysia (MYL), Argentina (ARG), and Nigeria (NER), with higher quantities of publications, followed by France (FRA), Spain (ESP), Thailand (THA), and China (CHN). The most extensive participation in this sector is mainly due to the Asian continent for this scenario.

In these subdivisions, several products are reported in which MDs were used for food formulations (Table 3). This group was classified as animal origin, bakery product, dairy frozen, fruit jelly, and vegetable origin according to careful analysis.

In **animal origin** products, MDs have been used to formulate mortadella [80], poultry pâté [81], and beef patties [85] low-fat; sausage [79] and grilled beef [83] with aromatic herbal; sodium-reduced sausages [26,78]; prebiotic sausage formulation [84], gluten-free chicken nugget [82], and honeydew honey [86].

In general, part of the studies have used MDs for formulations of probiotic products of animal origin, such as the formulation of a prebiotic sausage with inulin, konjac, and starch [84] or formulation of a poultry pâté with inulin and lentil flour [81]. Other uses of the MDs were to try to reduce the fat or sodium content in these products. Such as formulating a low-fat mortadella using beef meat, pork meat, and pork back fat [80], formulation of sodium-reduced lean sausages with fish oil seeking partial replacement of NaCl by KCl and sodium tripolyphosphate [26,78], and the formulation of low-fat beef patties with the partial animal fat replacement by cold-pressed grape seed oil and pomegranate seed oil [85].

Other less conventional applications of MDs were for formulations of natural sausages with the addition of aromatic herbs to slow the oxidation of lipids and control the spoilage bacteria in the food [79], or to evaluate the inhibitory effect of spices/herbs and their mixtures on the formation of heterocyclic amines and mutagenic activity of grilled beef by MDs [83]. Moreover, there was only one report for the development of gluten-free industrialized animal products, in which the authors formulated a gluten-free pasta based on three Thai rice flour for use in a frozen battered chicken nugget [82].

In **bakery products**, MDs have been used in functional milk [97], chocolate [94], pecan nut [101,102], and sponge [98] cake formulations; gluten-free formulation of bread [87,93] and cakes [88]; noodle [100] and rice bread [99] formulations; cake [89] and staple bread [95] formulation; cassava crackers enriched [96]; flour [91] and whole-food [90] blend formulation and cereal recipe [92].

Among the most frequent applications of MDs in bakery products are formulations of gluten-free products, such as the formulation of bread using sorghum flour, rice flour, and millet flour [87]; a gluten-free bread based on chickpea flour [93], and a gluten-free cake enriched with protein [88]. Other applications of MDs extend the incorporation of products with different purposes, such as inserting jujube flour in the sponge cake [98], the addition of tikhur starch as a substitute for semolina in the preparation of baked milk cake [97], formulation of chocolate cake with partial replacement of wheat flour with yacon and maca flour [94], formulation of cassava-fish crackers with a combination of ingredients such as cassava starch, high-quality cassava flour, and fish flour [96], formulation of rice noodles that include combinations of gelatinized corn starch, xanthan gum, and guar gum to improve the tensile strength of cooked noodles [100] and the formulation of a low-cost fiber-rich whole food from residues from the processing of orange juice [90].

In **dairy frozen**, the use of MDs was scarce. Among the applications, the use of MDs to optimize the combination of stabilizers (stone gum, carboxymethylcellulose, and guar gum) in the ice cream formulation [105], for the formulation of ice cream mix powder using milk protein, fat, sucrose, stabilizers, emulsifiers, and water [103], and formulation of antioxidants-rich ice cream containing aqueous extract rich in phenolic compounds and potential functional properties made of *Ilex paraguariensis*, *Melissa officinalis*, and *Cymbopogon citratus* [104].

In **fruit jelly,** the MDs were used for functional jelly formulations. Such as the formulation of a jelly enriched with apple puree, concentrated apple juice, and juice from sea-buckthorn berries added during the gelation of jelly [108] or the optimization of native Brazilian fruit jelly with jabuticaba, pitanga, and cambuci based on sensory and nutritional characteristics [106]. Other jelly formulations have been reported, such as jelly with Swazi indigenous fruits tincozi, tineyi, and umfomfo [107], jelly formulation of red fruit with blackberry, blueberry, and strawberry [109], and produce a pestil (fruit leather) from commercial pomegranate juice using xanthan gum, locust bean gum, and pregelatinized starch [110].

In **vegetable origin** products, the use of MDs went to tomato sauce formulated with tomato puree, onion puree, and extra virgin olive oil [115], elaboration of probiotic creamy sauce-based soymilk with okara flour fermented by *Lactobacillus acidophilus* [112]; oxidative stability of soybean oil with natural antioxidant from Tunisian aromatic plants [113,114] and replacement of salt NaCl by KCl and CaCl_2_ in tomato soup using electronic tongue and mixture design [111].

### 3.7. Application of Mixture Design in Pharmaceutical Health

Figure 4 shows the weight map for the pharmaceutical health variable. In scenario 1, only the lower right quadrant had greater weight for this variable. Unlike previous areas, Brazil (BRA) now does not maintain the apex of works and shares the space with India (IND). Other countries, such as Italy (ITA), Malaysia (MYL), and Morocco (MAR), also stand out. Canada (CAN), China (CHN), Iran (IRN), South Korea (KOR), Spain (ESP), United Kingdom (GBR), and United States (USA) had discreet participation. Moreover, Ecuador (ECU), Egypt (EGY), and France (FRA), among other countries represented by the same color scale, presented smaller quantities (*n* = 1). Afghanistan (AFG), Greece (GRC), Switzerland (CHE), and others represented by the same color scale did not have works.

According to Figure 4 of scenario 1, the Asian continent had greater participation in the pharmaceutical health area than the other continents. This greater participation is leveraged by India (IND), Malaysia (MYL), China (CHN), Iran (IRN), and South Korea (KOR), totaling *n* = 27 works. Then with the American continent, represented by publications from Brazil (BRA), Canada (CAN), and United States (USA), totaling *n* = 16. Moreover, the European continent represented intermediate participation, with the most representative countries being Italy (ITA), Spain (ESP), and the United Kingdom (GBR), totaling *n* = 11. In the African continent, only Morocco (MAR) presented a series of expressive publications (*n* = 4), while Oceania did not present any work for this application.

In scenario 2, the weight map was divided into two quadrants, one on the left side at the bottom of the map, represented by Morocco (MAR); and another on the right side at the top, represented by India (IND), Malaysia (MYL), Iran (IRN), and Italy (ITA). These countries were responsible for the largest number of publications, in which the Asian and European continents presented a significant number of publications in this scenario.

According to Table 4, the main applications of MDs in pharmaceutical health are for the development of novel products or the formulation of several drugs. This area was subdivided into five classes: drug formulation, encapsulation, extraction compounds, functional activity, and foams/or films-based.

In **drug formulation**, the greatest number of studies using MDs stands out. The application of this method in developing pharmaceutical products has been more explored than in other areas. In this sense, the researchers have used MDs to optimize the composition of formulations and simultaneously estimate the main effects and the interaction of all variables in a drug formulation for several diseases, such as osteoporosis, cardiovascular, cancer, AIDS, dermatological, tuberculosis, gastrointestinal, etc.

Among the main uses of MDs is the search for improvements in the physicochemical and rheological characteristics of products, or in the delivery system and bioavailability of drugs, or the development and formulation of nanostructured lipid carriers (NLCs), self-microemulsifying drug delivery system (SMEDDS), self-nanoemulsifying oily formulations (SNEOFs), solid self-nanoemulsifying oily formulations (S-SNEOFs), and self-nanoemulsifying lipidic nanomicelles systems (SNELS). Alternatively, in the preparation of micro, nanoemulsions, microencapsulation, and development of pharmaceutical nanosuspensions and some nanocrystal-based products.

Used to improve solubility of poorly soluble drugs [142]; medicated chewing gums [120]; bioactive dressings [128]; or tablets [121,139], oleogels [144,145], organogels for cardiovascular diseases [118], nanoparticles with amoxicillin trihydrate and thymol [131,134], novel excipients [129,136,146] and oils mixture with salicylic acid for ointment [143] formulation; itraconazole for oral [27,127], gel with loratadine [148], tablets of metformin [130], controlled release of ketoprofen [23] and excipients with finasteride [126] formulation; emulsion formulation of vitamins [119], of aceclofenac [122], of raloxifen [135], of aprepitant [123], of catechin [28], of darunavir [117], of lopinavir [116], of butenafine hydrochloride [132], of cathepsin K inhibitor [140], of telmisartan [138], of olmesartan medoxomil [137], of bovine serum albumin [141], of nelfinavir mesylate [124], of docetaxel [125], of nutraceutical products [147] and oxidative stability avocado oil [133].

The use of MDs has also been explored for the formulation of **encapsulants**. In this step, it is common to mix two or more components to improve specific properties of drugs, and a great way to evaluate the interaction between these encapsulating agents is through MDs. Among some applications, we can list the optimization of the ratio of materials to produce the cinnamon essential oil microcapsules by spray-drying with gum Arabic, maltodextrin, and inulin [149]; or to evaluate the effect of encapsulating agents (gum Arabic, modified starch Capsul™ and maltodextrin DE 5) on anthocyanin retention in microcapsules produced by spray-drying of raw pomegranate juice [150]. In addition, to formulate a bionanocomposites for entrapment of probiotic cells (*Bacillus coagulans*) using bacterial nanocellulose, pectin, and *Schizophyllum commune* [151]; or for the formulation of Turkish oregano extract microcapsules prepared by spray-drying using different concentrations of maltodextrin and gum Arabic as encapsulating agent [152]; or to check the performance of *Zingiber zerumbet* oil encapsulation [153]; or encapsulation using carbohydrate, protein, coconut oil mixtures on the viability of probiotic cells (*Lactobacillus bulgaricus*) during spray-drying [154].

The **functional activity** can have several classes, where antioxidant and antimicrobial are the activities most combined with MDs recently. MDs have been used to evaluate the synergistic effects of antioxidant activity on mixtures of the essential oil from *Apium graveolens* L., *Thymus vulgaris* L., *Coriandrum sativum* L., and essential oils from *Ocimum basilicum* L., *Origanum majorana* L., and *Rosmarinus officinalis* L. [162,163,165]. To maximize the antimicrobial activity of essential oils combined against *Escherichia coli* in milk [161] and *Salmonella typhimurium* [164], or to optimize strain mixture of *Lactobacillus* with the highest antimicrobial activity against common food-borne pathogenic bacteria (*Escherichia coli*, *Salmonella enteritidis*, *Listeria monocytogenes*, and *Bacillus cereus*) [166], the nematocidal activity of artemisia extract [167], maqui berry extract as an antioxidant/anti-inflammatory agent [168] and optimization of soy protein isolate, bovine whey protein and egg white protein hydrolysis with the protease Flavourzyme™ 500 L [169].

Certainly, the combination of essential oils generally expresses some interaction effect, and they can be synergistic, antagonistic, additive, or indifferent. In this sense, the MDs use has presented excellent perspectives for future studies to evaluate the effectiveness of several types of functional activities of these products [22].

MDs have been applied to **extract compounds** from various medicinal plants, such as anthocyanins and phenolic compounds from jabuticaba skin and seed, by mixing solvents [155]; to optimize the activity of total phenolic and flavonoids compounds from mixture herbs *Cnestis palala*, *Urceola micrantha*, *Labisia pumila*, and *Microporus xanthopus* [157]; optimize the solvent mixture to extract total phenolic content and antioxidant capacity of camu-camu (*Myrciaria dubia*) seeds [159]; optimize the solvent proportions for extraction of aporphine alkaloids from the leaves of *Unonopsis duckei* [160]; optimize the extraction of curcuminoids from turmeric using ethyl lactate, ethanol, and water under mild conditions [158]; and optimization of essential oil extraction from Pitanga (*Eugenia uniflora* L.) leaves with petroleum ether, *n*-hexane, methanol, and ethanol [156].

In this way, the use of MDs in the extraction of compounds is promising because it allows the identification of interactions between solvents of different polarities, in addition to the optimization of extraction techniques that can help in a multitude of applications, such as obtaining compounds of interest with greater yield and purity.

In **foams/films-based**, the MDs were used in the formulation of edible films/coatings composite with different proportions of pectin, alginate, and whey protein concentrate for evaluated for physicochemical characteristics on the final product [171] and formulation of foam of cassava starch, peanut skin, and glycerol to improve mechanical flexural mechanical properties and water absorption capacity [170].

### 3.8. Application of Mixture Design in Other Areas

Works classified in the category called “others” were selected because they appeared in the database set search using the previously defined keywords. Figure 4 shows the weight map for this variable. In scenario 1, the weight map presented a single quadrant with higher values in the lower direct part of the map. In this quadrant, all participation is again due to Brazil (BRA) with greater participation. Countries such as China (CHN) and Iran (IRN) had discreet participation. Portugal (PRT), Malaysia (MYL), and United States (USA), among others represented by the same color scale, presented smaller quantities (*n* = 1). Other countries, such as Belgium (BEL), Peru (PER), and Russia (RUS), among others, represented by the same color scale, did not present any work.

According to Figure 4 of scenario 1, the American continent had the largest participation in this area, with Brazil (BRA) being the main representative (*n* = 7). Although the Asian continent presents an expressive value (*n* = 10), no country belonging to that continent presented an expressive value (*n* > 2). In scenario 2, the weight map showed higher values in the right quadrant, represented by China (CHN) and Iran (IRN), with an *n* = 2. Countries such as Colombia (COL), Portugal (PRT), and Turkey (TUR), among others represented in the same color scale, presented *n* = 1. Moreover, countries separated on the left side of the map, such as Afghanistan (AFG), Argentina (ARG), Morocco (MAR), among others with the same color scale, presented a value of *n* = 0.

Table 5 presents a subdivision of the MDs’ possible applications that can be used within the area, and these subdivisions were classified as applications related to the area: animal, bioenergy, biology, and materials.

According to Table 5, MD research in **bioenergy** aims to optimize biodiesel synthesis from a blend of five different oils [181]; investigate the effects of cellulose, xylan, and lignin constituents on biomass pyrolysis characteristics and bio-oil composition [177]; production of biomethane using municipal sludge wastes, grease trap waste, and meat processing waste [179]; investigate the ability of the oleaginous yeast *Debaryomyces etchellsii* strain for lipid production for biodiesel manufacture using different agro-industrial wastewaters (cheese whey, expired soft drinks and fresh olive mill) as substrates [175]; investigate the effect of light wavelength on *Dunaliella salina* to increase microalgae lipid productivity for biodiesel production [176]; optimization of viscosity and density of refined palm oil-Melaleuca Cajuputi oil binary blends for a novel biofuel [180]; formulation of a vermicomposting in organic matters with dairy manure vermicompost, straw, and peat using MDs in cucumber seedling experiment to evaluate the compressed substrates [183]; optimization of concentrations of the solvents ethanol, acetone and water in the extraction of total phenolics present in cashew apple bagasse [178] and optimization of a method for the determination of metals from palm oil by flame atomic absorption spectrometry [182].

For the **materials** area, MDs have been used in glass formulation using iron phosphate base glass system that contained P_2_O_5_, Fe_2_O_3_, Al_2_O_3_, Na_2_O, and SO_3_ [29]; hydraulic oil formulation with different materials based on specific restrictions applying multiresponse optimization [189]; analysis of the effects of nano-oil additives (using ZnO, Si_3_N_4_ and carbon nanotubes) on the wear properties of AISI 4140 steel material [190]; formulate and examine the mechanical properties of cotton shell particles integrated into glass-fiber-reinforced polymer composites [187]; optimization of bitumen formulations using asphaltic residue, vacuum residue, and three aromatic extracts (by-products) from the refining process of base oils [188]; formulation of alkali-activated cement mortars incorporating glass powder, slag, and calcium aluminate cement [30]; and investigate the arsenic (V) removal from waters using synthetic minerals using synthetic poorly crystallized aluminum hydroxide, calcined layered double hydroxide, and two-line ferrihydrite [191].

In **biology** applications, MDs propose substitutes of bacteriological agar manufacture in terms of texture by using some reported gelling agents used by food and agroindustry [184], metabolomic fingerprint investigation of reference and crossed coffees [1], secondary metabolites from *Mikania laevigata* leaves [185] and development of a chromatography method to determine drugs of abuse [186].

In the **animal** area, MDs were used for the development of essential oil-based phyto-formulations to control the cattle tick *Rhipicephalus microplus* using a mixture of the cinnamon (*Cinnamomum zeylanicum*), cumin (*Cuminum cyminum*), and allspice (*Pimenta dioica*) [172]; formulation of functional foods for animals mixing complex extracts of *Rhodiola crenulata*, *Astragalus membranaceus*, and *Panax quinquefolius* [173]; and development of an analytical chromatography method for the simultaneous investigation of veterinary drugs in poultry litter [174].

### 3.9. Behaviors, Trends, and Perspectives of Mixture Design Applications

Regarding the use of mixture design, the main perspective raised was the development of healthier products, such as the formulation of symbiotic products, which combine probiotics and prebiotics in the form of synergism, and products that aim to reduce the content of sodium, fat, sugar, or gluten from food. Given the greater concern with food quality and improved quality of life, consumers have increasingly chosen these products, which have leveraged and heated the market in this sector. Moreover, there is a growing interest in formulations of free gluten, sugar, or lactose products, especially for celiac, diabetic, and lactose intolerance prevalence.

Another emerging field of research has been studies related to allergenic substances in foods from animal or plant sources that can cause an overreaction of the immune system. In our search, there were no reports about works on this theme. In this sense, MDs’ applications can be great tools for future formulations of these types of products.

We also observed promising prospects for studies to evaluate the substitution of chemical additives added to food and pharmaceutical products, such as antioxidants and synthetic stabilizers. Thus, the replacement by natural products such as herbs, fruits, and co-products from industrial processes are great paths to be followed, as they generate products with greater health benefits, add value to co-products, and have been the focus of intense research. Moreover, MDs are promising tools to assess the effects of synergistic interactions of functional activities, such as the antioxidant, antimicrobial and antiviral activity of essential oils and extracts of natural products.

Recently, the science and technology of the production of alcoholic beverages has been taken to another level. Consumers have shown a preference for functionalized drinks, such as the consumption of craft beers, one of the main reasons for this market’s leverage [22]. This trend has been growing and opens the possibility of using MDs for formulations of craft beers and other alcoholic beverages.

Though drug formulation has been the main use of MDs, this sector still has an overall capacity for research in nanotechnology, such as the development/formulation of novel NLCs, SMEDDS, SNEOFs, S-SNEOFs, SNELS, microemulsions, nanoemulsions, microencapsulation, nanosuspensions, and nanocrystal-based products.

Trends in the use of MDs in other areas are growing, some promising fields for the application of these tools have been reported, such as the reuse or use of sources from renewable raw materials and industrial waste, formulations of growth media, development of novel analytical methods, nanotechnology, novel materials such as graphene, silicene, perovskite and metal-organic frameworks (MOFs) with specific characteristics for many applications.

Another general trend is the use of MDs to solve problems with multiple responses: restrictions of maximum, minimum, and target values for the responses that can be applied when seeking a formulation [5,6,22]. The strategy most used to optimize multiple responses makes use of the desirability function (D), proposed by Deringer and Suich [192], and other methods such as overlaid contour plots, constrained nonlinear optimization, and a Bayesian approach, which has also been employed [31]. From a practical point of view, multiresponse optimization makes the use of MDs more attractive. Among some promising examples of the application of the desirability function (D) present in the literature, we can mention the multiresponse optimization of the oxidative stability of biodiesel from natural [19] and synthetic [21] antioxidants, or a multiresponse optimization of the efficiency and cost of synthetic antioxidants added to biodiesel [6], or biodiesel formulation [5], or a multiresponse optimization of the effects of adding spices with antioxidant compounds in craft beer [22].

### 3.10. Statistical Packages Used for Mixture Design Applications

Most statistical packages can be used to conduct classic DoE; however, MDs require specialized software. Among the software most used by researchers, we highlight the design expert (Stat-Ease, Inc., Minneapolis, MN, http://www.statease.com/ accessed on 5 January 2021), present in approximately 46% of the evaluated works, followed by Statistica (StatSoft Onc., South America, Tulsa, OK, USA, https://www.statsoft.de/en/home accessed on 5 January 2021), with 22%, the Minitab (Minitab Inc., State College, PA, USA, https://www.minitab.com/ accessed on 5 January 2021) with 9%, the Statgraphics (Statistical Graphics Corporation, The Plains, Virginia, VA, USA, www.statgraphics.com accessed on 5 January 2021) with 5%, the Matlab (The Mathworks Inc., Natick, MA, USA, https://www.mathworks.com/ accessed on 5 January 2021) with 4%, and the JMP (SAS Institute, Inc., Cary, NC, USA, https://www.jmp.com accessed on 5 January 2021) with 3%.

We emphasize that all the mentioned software is not available for free. Some free software options have also been reported less frequently. Among the most common free software, we had R (R Core Team, Vienna, Austria, www.r-project.org accessed on 5 January 2021) present in 4% of the works. This software has exclusive MD packages, among the most widespread, we found the package “mixexp” and “qualityTools”. Another less frequent free software was Chemoface software (Brazil, BA, Salvador), present in 4% of the papers. Chemoface is a MATLAB stand-alone application. To run it, you must install MATLAB Compiler Runtime (MCR), which is freely available in https://www.ufla.br/chemoface/ accessed on 5 January 2021.

## 4. Conclusions

The data presented in this meta-analysis indicated the MDs’ capacity and applicability for the development or formulation of novel products applied in the area of food, beverage, and health science. We also emphasize the applicability of MDs in food and beverage science to formulate foods or beverages with specific characteristics that provide additional health benefits, such as functional and nutraceutical products. In pharmaceutical health, the MDs showed applicability for developing or formulating novel drugs for various diseases. In addition, they emphasize the importance of Brazil for the development of products applied in the area of food and beverage science using MDs and the importance of India, Brazil, Malaysia, and Italy in the pharmaceutical health area.

Furthermore, we present a novel approach to meta-analysis studies through self-organizing maps, as successfully applied previously in our study [34]. This approach allows processing metadata using non-conventional statistical methods that verify cluster trends and similarities. However, this preliminary approach can be extended to other sources or areas of food science. In this sense, this work can be seen as a guide for novel meta-analysis studies.

## Figures and Tables

**Table 1:** Abbreviated country names according to the United Nations.

Countries	UN	Countries	UN	Countries	UN	Countries	UN
Afghanistan	AFG	Ecuador	ECU	Japan	JPN	Serbia	SRB
Argentina	ARG	Eswatini	SWZ	Lithuania	LTU	Singapore	SGP
Australia	AUS	Ethiopia	ETH	Malaysia	MYL	South Africa	ZAF
Belgium	BEL	Finland	FIN	Morocco	MAR	South Korea	KOR
Brazil	BRA	France	FRA	Mexico	MEX	Spain	ESP
Bulgaria	BGR	Germany	DEU	New Zealand	NZL	Sweden	SWE
Cameroon	CMR	Greece	GRC	Nigeria	NER	Switzerland	CHE
Canada	CAN	Hong Kong	HKG	Pakistan	PAK	Tanzania	TZA
Chile	CHL	India	IND	Peru	PER	Thailand	THA
China	CHN	Indonesia	IDN	Poland	POL	Tunisia	TUN
Colombia	COL	Iran	IRN	Portugal	PRT	Turkey	TUR
Czech Republic	CZE	Ireland	IRL	Russian	RUS	United Kingdom	GBR
Egypt	EGY	Italy	ITA	Saudi Arabia	SAU	United States	USA

**Table 2:** Application of mixture design in the beverage.

Papers	*n*_ref_	References for Data Analysis
Application in Beverage	37	Refs.
Alcoholic beverage	1	[43]
Milk-based beverages	14	[44,45,46,47,48,49,50,51,52,53,54,55,56,57]
Several beverages	9	[25,58,59,60,61,62,63,64,65]
Several juices	13	[24,66,67,68,69,70,71,72,73,74,75,76,77]

**Table 3:** Application of mixture design in food.

Papers	*n*_ref_	References for Data Analysis
Application in Food	39	Refs.
Animal origin	10	[26,78,79,80,81,82,83,84,85,86]
Bakery product	16	[87,88,89,90,91,92,93,94,95,96,97,98,99,100,101,102]
Dairy frozen	3	[103,104,105]
Fruit jelly	5	[106,107,108,109,110]
Vegetable origin	5	[111,112,113,114,115]

**Table 4:** Application of mixture design in pharmaceutical health.

Papers	*n*_ref_	References for Data Analysis
Application in Pharmaceutical Health	59	Refs.
Drug formulation	36	[23,27,28,116,117,118,119,120,121,122,123,124,125,126,127,128,129,130,131,132,133,134,135,136,137,138,139,140,141,142,143,144,145,146,147,148]
Encapsulation	6	[149,150,151,152,153,154]
Extraction compounds	6	[155,156,157,158,159,160]
Functional activity	9	[161,162,163,164,165,166,167,168,169]
Foams/films-based	2	[170,171]

**Table 5:** Application of mixture design in other areas.

Papers	*n*_ref_	References for Data Analysis
Application in Other Areas	23	Refs.
Animal	3	[172,173,174]
Bioenergy	9	[175,176,177,178,179,180,181,182,183]
Biology	4	[1,184,185,186]
Materials	7	[29,30,187,188,189,190,191]

## Data Availability

The data presented in this study are available on request from the corresponding author.

## Supplementary Materials

The following are available online at https://www.mdpi.com/article/10.3390/foods10081941/s1, Supplementary Materials: MDs types. The theoretical and mathematical foundations of the SOM algorithm.
