# Supplementary material for: Recent Applications of Mixture Designs in Beverages, Foods, and Pharmaceutical Health: A Systematic Review and Meta-Analysis
Source: Foods. 2021 Aug 20;10(8):1941. doi: 10.3390/foods10081941 (PMC8391317; doi:10.3390/foods10081941)
Supplement: Supplementary file 1 [file foods-10-01941-s001.zip › foods-1322160-SI.pdf]

Supplementary material: The theoretical and mathematical foundations of the SOM algorithm.

Simplex Lattice Design (SLD)

SLD are used to study the effects of the mixture compounds on the response variable. In which it provides an equally spaced distribution of points over the factor space [1–4]. A {q, m} simplex lattice design for q components consists of points defined by the following coordinate settings, where the proportions assumed by each component take the m + 1 values, equally spaced values from 0 to 1. Component levels are obtained through Eqn. (1):

$$x_i = 0, \frac{1}{m}, \frac{2}{m}, \dots, 1 \quad i = 1, 2, \dots, q$$

the design space consists of all possible combinations (mixtures) of the proportions used, and m is called the degree of a lattice. The number of points (N) of an SLD is given by Eqn. (2) [1,2].

$$N = \frac{(q+m-1)!}{m!\,(q-1)!}$$

Some SLD plans are shown in Figure S1.

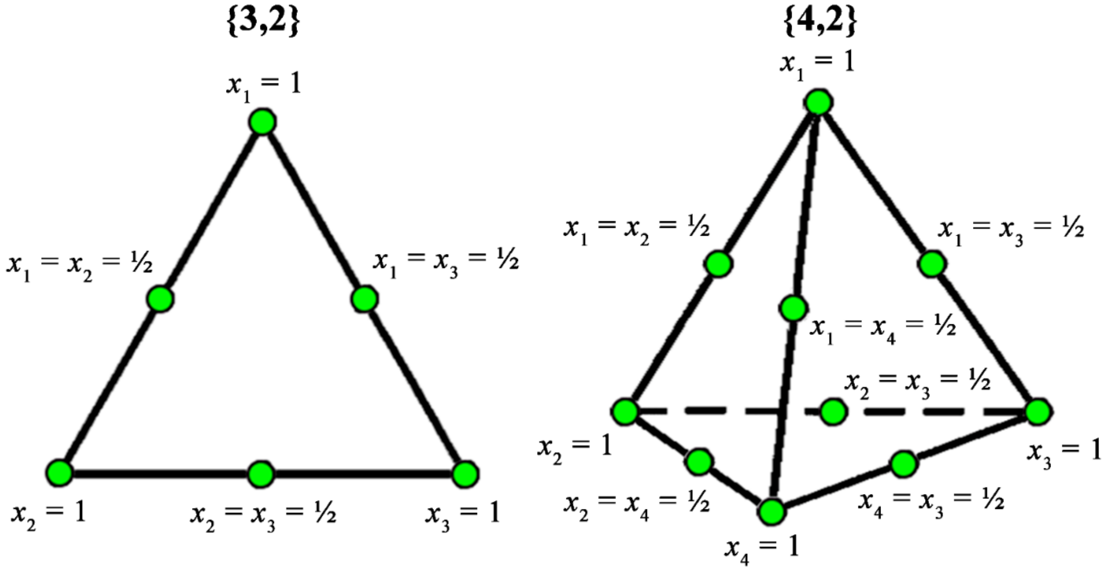

Figure S1. – Some {*q*, *m*} lattices design for a {3, 2} its design space has 6 points (left) and for a {4, 2} design space has 10 points (right).

Simplex-Centroid Design (SCD)

An alternative to the simplex model was introduced by Scheffé [5] named with simplex-centroid design (SCD), in which only the centroid points are included [1,2,4–6]. In an SCD for q-components, there are 2^{q-1} points, corresponding to the q permutations of (1, 0, 0, ...,0), the $\binom{q}{2}$ permutations of (½, ½, 0, ..., 0), the $\binom{q}{3}$ permutations of (⅓, ⅓, ⅓, 0, ..., 0), and the overall centroid $(1/_q, 1/_q, \dots, 1/_q)$. If the centroid degree is set to (m < q), the total number of runs will be $\binom{q}{1} + \binom{q}{2} + \cdots + \binom{q}{m}$ [2,5].

Most of the experimental runs in the SCD occur on the boundary of the region and experimental region, consequently including only the $q - 1$ of the q components. It is usually

desirable to augment the simplex lattice or simplex centroid with additional points in the interior of the region where the blends will consist of all q mixture components [1,2,7].

Some SCD plans are shown in Figure S2.

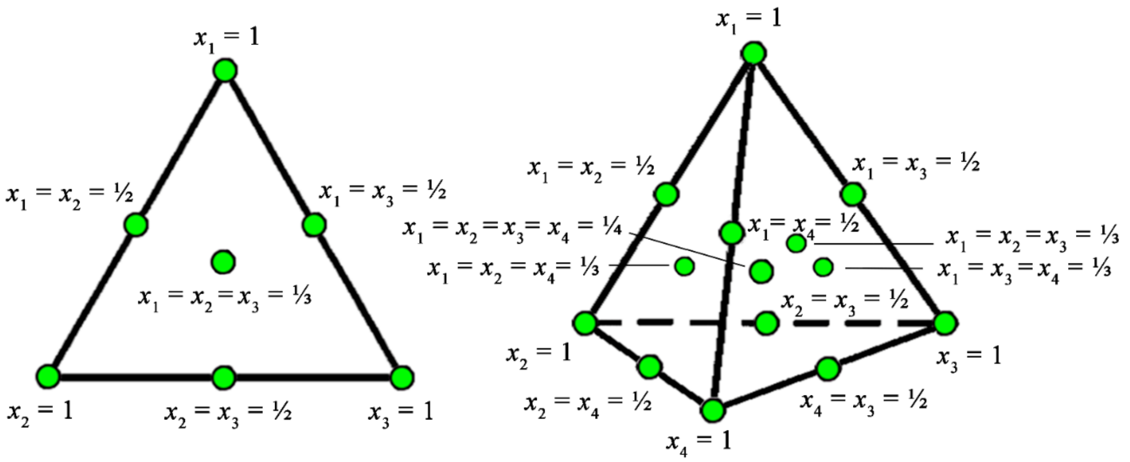

Figure S2. – Some simplex-centroid design {3, 2} for 3 components with 7 points (left) and {4, 2} 4 components with 14 points (right).

Simplex Axial Design (SAD)

SAD was developed by [8] for use in screening experiments or measuring individual component effects, where most points are positioned inside the simplex and are mixtures of q components [4]. The axis of component i is the imaginary line or ray extending from the base point $x_i = 0$, $x_j = 1/(q-1)$ for all $j \neq i$ to the opposite vertex, where $x_i = 1$, $x_j = 0$ for all $j \neq i$. All points in an axial design are positioned on the axes of the factor space. The base point will always lie at the centroid of the $(q-2)$-dimensional boundary of the simplex that is opposite to the vertex $x_i = 1$, $x_j = 0$ for all $j \neq i$. In this design, all points are in the axial; the simplest form of axial design is one in which points are positioned equidistant from the overall centroid $\left(1/q, 1/q, \ldots, 1/q\right)$. Points located in the middle from the general centroid to the vertex are called axial points/mixtures [1,2,4].

A SAD plan is shown in Figure S3.

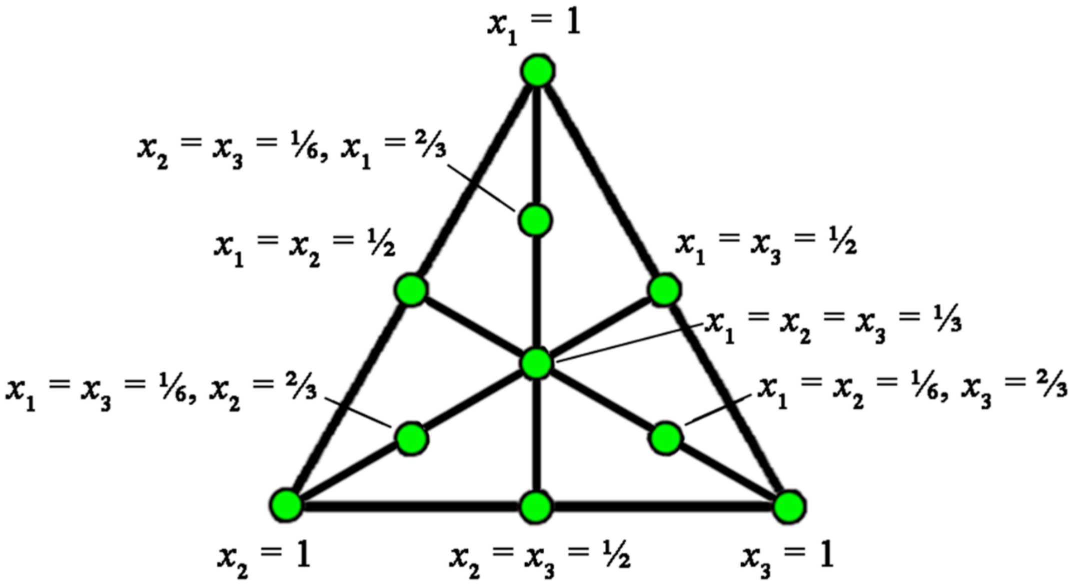

Figure S3. – Simplex axial design for 3 components has 10 points.

Extreme Vertices Designs (EVD)

EVDs are types of MDs that include only a subpart or a smaller space inside the simplex [4,9–11]. This condition occurs when additional constraints of the form of upper and/or lower limits are established for the proportions of the components located within the simplex. The general form of the constraints mixing problem is $x_1 + x_2 + ... + x_q = 1$ with $l_i \leq x_i \leq u_i$, for $i = 1, 2,...,$ q, where l_i is the lower limit of the i-th component and u_i is the upper limit for the i-th component, with $l_i \geq 0$ and $u_i \leq 1$ [2]. Three forms of the constrained mixture problem are most widely used, being D-optimality (determinant), G-optimality (global), and I-optimality (integrated). The D-optimal design focuses on estimating the best possible model coefficients; this criterion results in maximizing of the parameter estimates, the G-optimal design minimizes the maximum value of prediction variance, and the I-optimal design seeks to minimize the average scaled prediction variance over the design region [12,13].

To the EVD with lower limits, the perception of pseudocomponents is applied [1]. Defined in the Eqn. (3).

$$x_i' = \frac{x_i - l_i}{\left(1 - \sum_{j=1}^{q} l_j\right)} \quad \textbf{(7)}$$

with $\sum_{j=1}^{q} l_j < 1$. The pseudocomponents are transformed into formulations for the original components by reversing the transformation to have again $\sum_{i=1}^{q} x_i' = 1$, thus being able to use simplex plans. To obtain the value of x_i for a given x_i', it is necessary to manipulate the Eqn. (3), obtaining Eqn. (4).

$$x_i = l_i + \left(1 - \sum_{j=1}^{q} l_j\right) x_i' \quad \textbf{(8)}$$

For this situation, the feasible region of the simplex is no longer a triangular simplex and has developed an irregular shape [1].

An EVD plan is shown in Figure S4.

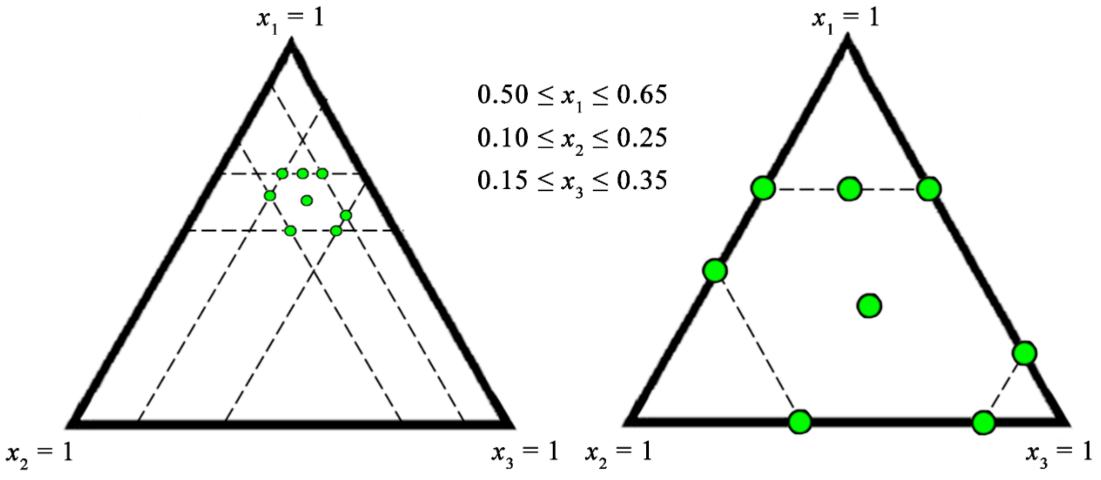

Figure S4. – Extreme vertices designs for 3 components has the following constraints (left) and in terms of pseudocomponents (right).

SOM algorithm

SOM-type ANN routine developed was used according to the algorithm described in Haykin [14] and was applied using the Matlab software routine.

The function chosen to represent the topological neighborhood in Eqn. (5).

$$h_{j,i} = \exp(-d_{j,i}^2 / 2\sigma^2) \quad \textbf{(5)}$$

where σ is the effective radius of the topological neighborhood, and $\boldsymbol{d}_{j,i}$ is the lateral distance between the "winning neuron" i and the excited neuron j. Over the training epochs, there is a reduction in the neighborhood's size due to an exponential decay, Eqn. (6).

$$\sigma = \sigma_0 \exp(-n/\tau_1) \quad n = 0,1,2,\ldots \tag{6}$$

where σ_0 is the effective radius in the initialization of the algorithm, τ_1 is the time constant, with $\tau_1 = 1000/\log \sigma_0$ being recommended, and n is the number of training epochs.

During the adaptive process, it is necessary that the synaptic weight vector ($\mathbf{w}_j$) of the j neuron in the grid be modified in relation to the input vector $\mathbf{x}$. The modification process is a modification of the Hebb postulate of learning, described by Eqn. (7).

$$\mathbf{w}_j(n+1) = \mathbf{w}_j(n) + \eta(n) h_{j,i(x)}(n) \left(\mathbf{x} - \mathbf{w}_j(n)\right) \tag{7}$$

where $\eta(n)$ is the learning rate, which, as shown, is variable and decreases during the training epochs, n. The learning rate decrease may be modeled by an exponential decay, as described in Eqn. (8). In this equation, η_0 is the initial learning rate, and τ_2 is another time constant; the recommended values are respectively 0.1 and 1000:

$$\eta(n) = n_0 \exp(-n/\tau_2) \tag{8}$$

Table S2

In this study, all 52 countries reported were considered in the analysis, where n represents the sum of scientific papers using MDs in each continent, see **Table S2**.

Table S2. – Scientific papers published between 2016 and 2020 using Mixture Designs by continent.

Continents	Beverage	Food	Health	Others	Sum
America*	21	17	20	10	68
Africa	4	6	9	3	22
Asia	14	13	31	10	68
Europe	7	12	18	4	41
Oceania	2	0	0	1	3
Sum	48	48	78	28	**202**
Brazil	13	11	11	7	42

*Brazil included in the sum.

References

1. Calado, V.; Montgomery, D.C. *Planejamento de Experimentos Usando o Statistica*; Editora E-papers: Rio de Janeiro, Brazil, 2003; ISBN 8587922831.
2. Montgomery, D.C. *Design and Analysis of Experiments*, 6th ed.; John Wiley and Sons: New York, NY, USA, 2005.
3. Scheffé, H. Experiments with Mixtures. *J. R. Stat. Soc. Ser. B* **1958**, *20*, 344–360, doi:10.1111/j.2517-6161.1958.tb00299.x.
4. Cornell, J.A. *Experiments with Mixtures: Designs, Models and the Analysis of Mixture Data*, 3rd ed.; Wiley: New York, NY, USA, 2002; ISBN 9780471393672.
5. Scheffé, H. The Simplex-Centroid design for experiments with mixtures. *J. R. Stat. Soc. Ser. B Stat. Methodol.* **1963**, *25*, 235–251, doi:10.1111/j.2517-6161.1963.tb00506.x.
6. Hasan, T.; Ali, S.; Ahmed, M. Shrinkage simplex-centroid designs for a quadratic mixture model. *J. Ind. Eng. Int.* **2018**, *14*, 87–93, doi:10.1007/s40092-017-0210-1.
7. Barros Neto, B.; Scarminio, I.S.; Bruns, R.E. *Como Fazer Experimentos: Pesquisa e Desenvolvimento na Ciência e na Indústria*; 4th ed.; Bookman: Porto Alegre, Brazil, 2010; ISBN 8577807134.

8. Cornell, J.A. Some comments on designs for cox's mixture polynomial. *Technometrics* **1975**, *17*, 25–35, doi:10.1080/00401706.1975.10489267.
9. Myers, R.H.; Montgomery, D.C.; Anderson-Cook, C.M. *Response Surface Methodology: Process and Product Optimization Using Designed Experiments*, 2nd ed.; John Wiley & Sons, Inc.: New York, NY, USA, 1995; ISBN 978-0-471-58100-0.
10. McLean, R.A.; Anderson, V.L. Extreme vertices design of mixture experiments. *Technometrics* **1966**, *8*, 447–454, doi:10.1080/00401706.1966.10490377.
11. Piepel, G.F. Defining consistent constraint regions in mixture experiments. *Technometrics* **1983**, *25*, 97–101, doi:10.1080/00401706.1983.10487824.
12. Buruk Sahin, Y.; Aktar Demirtaş, E.; Burnak, N. Mixture design: A review of recent applications in the food industry. *Pamukkale Univ. J. Eng. Sci.* **2016**, *22*, 297–304, doi:10.5505/pajes.2015.98598.
13. Piepel, G.F. 50 years of mixture experiment research: 1955–2004. In *Response Surface Methodology and Related Topics*; World Scientific: Singapore, 2006; pp. 283–327.
14. Haykin, S. *Neural Networks: A Comprehensive Foundation*; Prentice Hal: New York, NY, USA, 2001; ISBN 978-0-02-352761-6.
